# Induction of PrMADS10 on the lower side of bent pine tree stems: potential role in modifying plant cell wall properties and wood anatomy

**DOI:** 10.1038/s41598-019-55276-7

**Published:** 2019-12-12

**Authors:** Nicolás Cruz, Tamara Méndez, Patricio Ramos, Daniela Urbina, Andrea Vega, Rodrigo A. Gutiérrez, María A. Moya-León, Raúl Herrera

**Affiliations:** 1 Instituto de Ciencias Biológicas, Universidad de Talca. Av. Lircay s/n, Talca, Chile; 2 FONDAP Center for Genome Regulation. Millennium Institute for Integrative Systems and Synthetic Biology. Departamento de Genética Molecular y Microbiología. Facultad Ciencias Biológicas, P. Universidad Católica de Chile, Alameda 340, Santiago, Chile; 3 Facultad de Ciencias Ambientales, Universidad Técnica Estatal de Quevedo, Quevedo, Ecuador; 4 Núcleo Científico Multidisciplinario-DI, Universidad de Talca. Av. Lircay s/n, Talca, Chile

**Keywords:** Transcriptomics, Plant molecular biology

## Abstract

The molecular mechanisms underlying inclination responses in trees are unclear. In this study, we identified a MADS-box transcription factor differentially expressed early after inclination in the stems of *Pinus radiata* D. Don. *PrMADS10* has a CDS of 582 bp and encodes a group II MADS-box transcription factor. We measured highest accumulation of this transcript on the lower side of inclined pine stems. In an effort to identify putative targets, we stably transformed *Arabidopsis thaliana* with a *35S::PrMADS10* construct. Transcriptome analysis revealed 1,219 genes differentially-expressed, with 690 and 529 genes up- and down-regulated respectively, when comparing the transgenic and wild-type. Differentially-expressed genes belong to different biological processes, but were enriched in cell wall remodeling and phenylpropanoid metabolic functions. Interestingly, lignin content was 30% higher in transgenic as compared to wild-type plants consistent with observed changes in gene expression. Differentially expressed transcription factors and phenylpropanoid genes were analyzed using STRING. Several MYB and NAC transcription factors showed interactions with genes of the phenylpropanoid pathway. Together, these results implicate *PrMADS10* as a regulatory factor, triggering the expression of other transcription factors and genes involved in the synthesis of lignin.

## Introduction

The loss of verticality in trees triggers morphological changes in the cell wall, which affect wood quality. The underlying molecular response involves the expression of particular genes, calcium signaling, and the synthesis of hormones like auxins and ethylene^1–7^.

Remodeling of the secondary cell wall (SCW) is regulated by two large families of transcription factors (TFs): R2R3-MYB and NAC^5,8–13^. For example, the synthesis of the major secondary wall components such as cellulose, hemicelluloses and lignins, was regulated by TFs expressed in differentiating xylem of *Eucalyptus*. *EgMYB1* acts as repressor^14^ and *EgMYB2* as activator^15^ in this process. Both regulate the entire development of SCW, acting therefore as a second level of “master switch” due to the fact that NAC TFs like SND1 (Secondary Wall-associated NAC Domain Protein^16^) is at the top level of the hierarchical network. Apparently, *AtMYB46*, the orthologue of *EgMYB2* is a direct target of SND1^8^. Similarly, the expression of two MYB TFs from *Populus trichocarpa* are also activated by *Ptr*WND2, which is an orthologue of SND1 in *Populus trichocarpa*^17^.

*PtrWNDs* are expressed in stem structure of *P. trichocarpa*^18^ which could be either fiber- or vessel-specific expression. Ohtani^19^ described 12 *PtrWND* genes that redundantly modulate the differentiation of vessels and fiber cells during xylem formation. These TFs modulate the expression of genes involved in SCW formation and programmed cell death, and also other TFs^18,19^.

Other NACs and MYBs which participate in the regulation of SCW dynamics have also been identified both in Arabidopsis and trees. These include *PtrMYB28*^9^ and *PtrMYB1*^20^ potential orthologues of lignin-specific MYB activators^21^, as well as *PtrNAC118, 122, 128* and 129 from *P. trichocarpa*^22^ which are potential orthologues of Xylem NAC Domain 1 (XND1), a transcriptional repressor that regulates the expression of genes involved in programmed cell death and SCW formation^23^. Interestingly, the overexpression of SND2 is associated to particular phenotypes in woody and herbaceous stems^24^.

Other TF families, apart from NAC and MYB, have been involved in regulating the biosynthesis of SCW. For example, KNOTTED1-Like Homeodomain Protein7 targeted directly SND1^25^. This protein negatively regulates secondary wall biosynthesis in poplar and *Arabidopsis*, functioning as a negative feedback loop that represses metabolically SCW formation, and maintaining homeostasis^26^. Another TF, *Nt*LIM1 regulates lignin biosynthesis pathway in tobacco and *E. camaldulensis* transformants^27,28^.

A partial sequence corresponding to a MADS-box TF was identified in a subtractive suppressed hybridization library (SSH) of one-year old radiata pine seedlings exposed to inclination^29^. The transcription factor MADS-box gene belongs to a highly-conserved multigene family previously identified in a wide range of eukaryotic genomes^30^. These proteins are major regulators of plant development, and the expression of the gene has been described in roots, stems, abscission zones, leaves, developing ovules and embryos^31–33^. Also, the formation of higher order MADS-box complexes is a means by which they obtain their diverse functions^34,35^. These TFs are known to be involved in flowering^36^, ripening processes in fruit^37,38^ and anthocyanin biosynthesis^39–41^.

MADS-box proteins share a highly-conserved DNA-binding domain, with a length of 56–60 amino acid residues. MADS-box proteins recognize a CC(A/T)_6_GG DNA sequence known as the CArG-box element, and the functional role is performed as a protein dimer^42,43^. Even if most reports have related their role in flowering, several authors have shown that MADS-box genes are expressed in differentiating primary/secondary xylem and phloem during wood formation in poplar^44^. In eucalyptus, MADS-box TFs have been detected in vegetative tissues^45^, and xylem tissue of white spruce^6^. Nine MADS-box genes have been identified in *P. radiata* (PrMADS 1 to 9), which have been detected in vegetative outbreak, floral organs and roots, all of them orthologues of TOMATO MADS3 (TM3). PrMADS 4 to 9 were more abundantly-expressed in young flowering tissue than in adult tissue^46^ and are members of the TM3 clade. But *PrMADS1* is orthologue to AGL2 clade and, *PrMADS2*, *PrMADS3* are members to AGL6 clade, playing a possible roles in regulation of reproductive development^47–49^.

Functional analysis of TFs from trees is not an easy task, so the use of the model plant *Arabidopsis thaliana* can help to provide clues concerning their functional role. For example, the expression of *Populus tremuloides MADS-box* 3 and 4 (PTM3/4) genes in Arabidopsis shows that they take part in floral development^50^, whilst, PTM5 is involved in vegetative development^51^. The constitutive expression of two *MADS-box* TFs members in Arabidopsis, *SHP1* and *SHP2* genes (previously known as *AtAGL1* and *AtAGL5*, respectively), promotes the lignification of cells adjacent to reproductive organs^52^.

The present report shows the identification and characterization of *PrMADS10*. This *MADS-box* gene has greater expression levels in the stems of inclined radiata pine, tissues which also rapidly-accumulate lignin^29^. Is *PrMADS10* a regulator for the synthesis of lignin? What other genes are modulated by the over expression of *PrMADS10*? These questions were answered by heterologously-overexpressing full length *PrMADS10* in Arabidopsis, carrying out a microarray assay and performing a MapMan^53,54^ analysis to obtain a metabolic overview of differentially expressed genes.

## Results

### Sequence and phylogenetic analysis of *PrMADS10*

**Figure 1:**
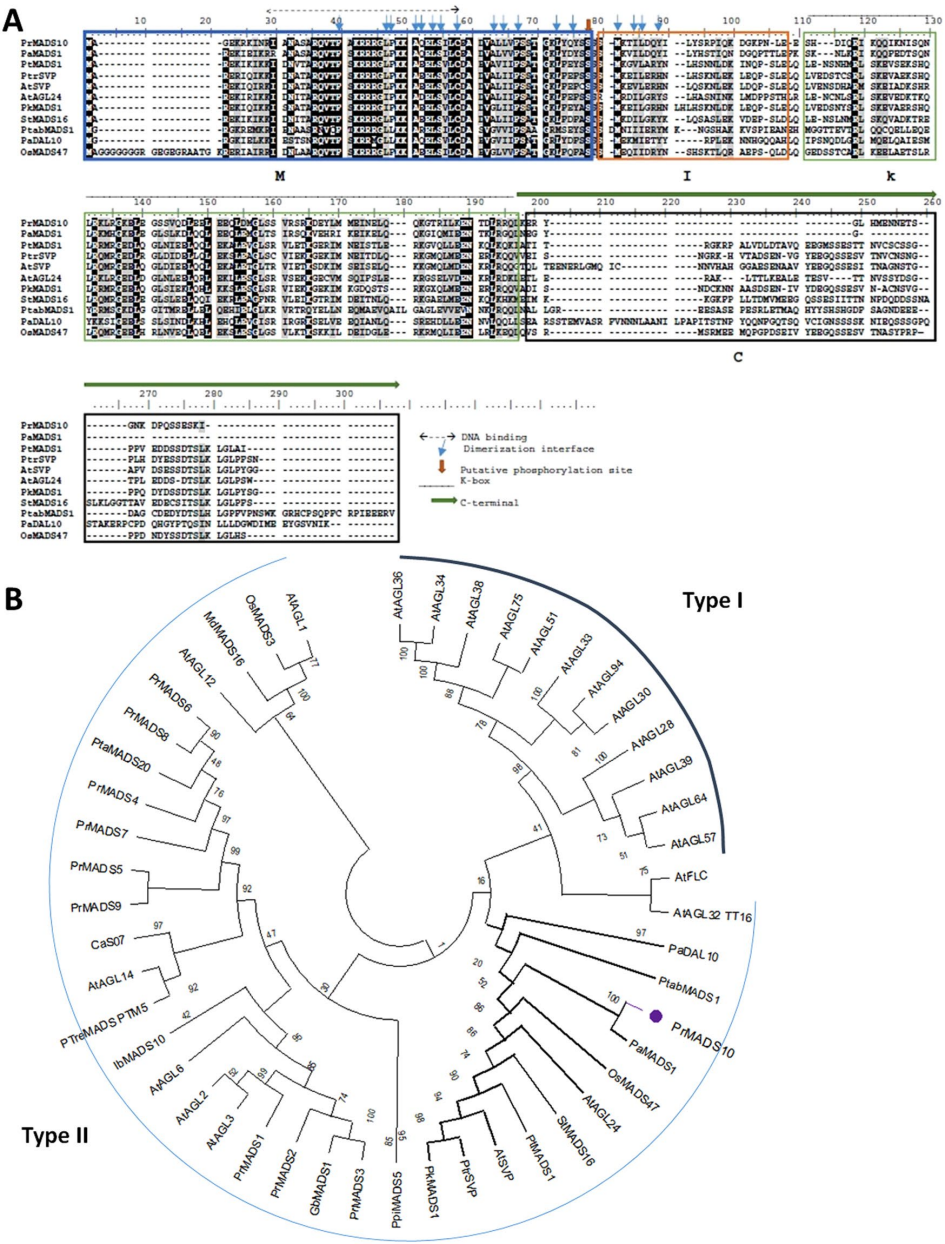
Sequence analysis of the deduced PrMADS10 protein from radiata pine with other MADS-box proteins. (**A**) Multiple alignment of the deduced PrMADS10 sequence with *Arabidopsis thaliana*, *Oryza sativa*, *Paulownia kawakamii, Picea abies, Pinus tabuliformis, Populus trichocarpa*, and *Solanum tuberosum* MADS-box TFs was performed using Clustal W and BioEdit Sequence Alignment Editor v7.0 software. Gaps are indicated by dashes, letters with a black background are identical amino acids, and letters with a gray background are similar amino acids. The box indicate M, I, K and C domains shared between MADS-box proteins, the double arrow and lines are DNA binding, thin arrow is dimerization interface; the filled arrow is a putative phosphorylation site. (**B**) Phylogenetic analysis was performed using MEGA X software, with Neighbor-joining, bootstrap consensus tree inferred from 10000 replicate. The evolutionary distances were computed using the Poisson correction method and are in the units of the number of amino acid substitutions per site. This analysis involved 48 amino acid sequences: *Arabidopsis thaliana* AtAGL1 (AAA32730), AtAGL2 (BAC43207), AtAGL3 (NP_849930), AtAGL6 (NP_182089), AtAGL12 (AEE35216), AtAGL14 (AEE83062), AtAGL24 (AEE84922), AtAGL28 (AEE27300), AtAGL30 (AEC05661), AtAGL32 (NP_974823) AtAGL33 (AEC07824), AtAGL34 (AED93593), AtAGL36 (AED93581), AtAGL38 (AEE34356), AtAGL39 (AED93653), AtAGL51 (AEE82144), AtAGL57 (AEE74037), AtAGL64 (AEE31158), AtAGL75 (AED94653), AtAGL94 (AEE34947), AtSVP (NP_179840), AtFLC (NP_196576), *Coffea arabica* CaS07 (ADU56825), *Ginkgo biloba* GbMADS1 (AIC79629), *Ipomoea batatas* IbMADS10 (ABD66305), *Malus domestica* MdMADS16 (BAG48168), *Oryza sativa* OsMADS3 (Q40704), OsMADS47 (Q5K4R0), *Paulownia kawakamii* PkMADS1 (AAF22455), *Picea abies* PaDAL10 (AAQ13443), *Pinus pinaster* PpiMADS5 (est_pipn_28509135_001R), *Pinus radiata* PrMADS1 (AAD09206), PrMADS2 (AAD09207), PrMADS3 (AAB58907), PrMADS4 (AAB80807), PrMADS5 (AAB80808), PrMADS6 (AAB80809), PrMADS7 (AAB80810), PrMADS8 (AAC27353), PrMADS9 (AAC80806), PrMADS10 (AKC96434), *Pinus tabuliformis* PtabMADS1 (AJP06319), *Pinus taeda* PtaMADS20 (est_pita_11126880, est_pita_11604453, est_pita_9457518), *Prunus avium* PaMADS1 (ABW82563), *Populus tomentosa* PtMADS10 (AAR92206), *Populus tremuloides* PtreMADS (AAP46287), *Populus trichocarpa* PtrSVP (XP_002310310), *Solanum tuberosum* StMADS16 (AAV65504).

*PrMADS10* full-length cDNA sequence was obtained using a partial EST sequence as template from the SSH library^29^, followed by 5′- and 3′-RACE-PCR. The sequence of 943 bp long contains 111 and 250 bp of 5′- and 3′-UTRs, respectively. *PrMADS10* has a CDS of 582 bp, encoding a deduced protein of 193 amino acids and 22 kDa (pI 9.42; GenBank accession number, KM887510; Fig. 1A). The predicted PrMADS10 protein has the typical conserved structural features of MADS-box TFs and possesses a MIKC type protein structure (Fig. 1A). A phylogenetic analysis was performed with 48 MADS-box amino acid sequences, including proteins from poplar, pine and spruce. PrMADS10 is classified in group II (MIKC^c^) according to MADS sub classification, and very close to orphans genes like AtSVP and StMADS16, genes that are expressed in vegetative tissue (Fig. 1B).

### *PrMADS10* transcripts are preferentially accumulated in pine stems and its protein has nuclear localization

**Figure 2:**
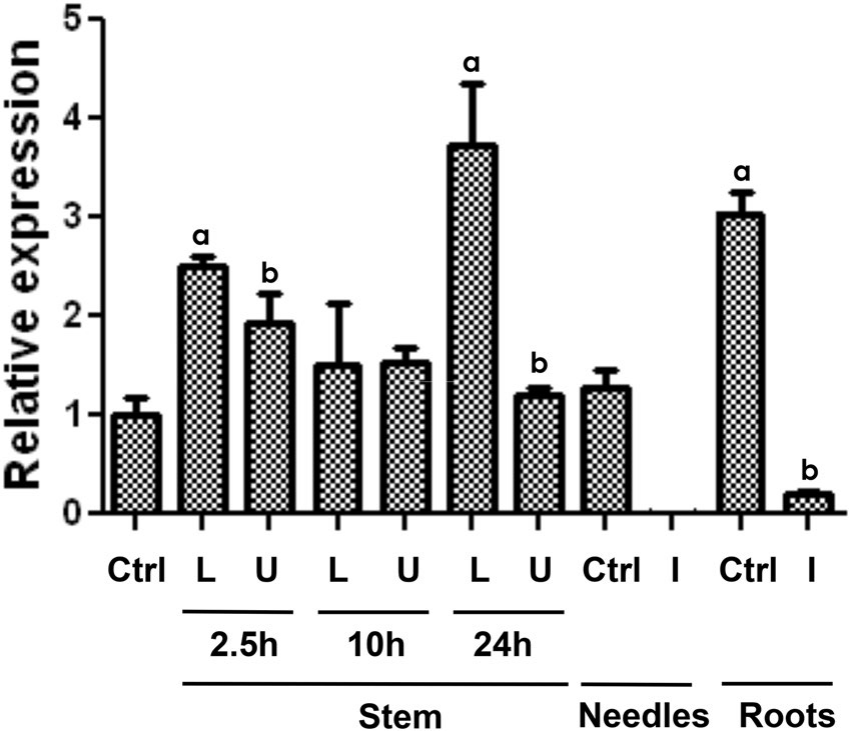
Transcripts levels of *PrMADS10* in young radiata pine seedlings after inclination stimuli. Stem samples were taken at different times of inclination either on the lower stem side (L) or upper stem side (U) of the stem. Roots and needles were obtained from the same inclined (I) seedlings. Ctrl (control) means non-inclined seedlings. Data correspond to mean ± SE of three biological replicates and different letters indicate statistical differences (*p* ≤ 0.05).

RT-qPCR analysis shows a higher transcript accumulation of *PrMADS10* in the lower side of inclined stems, reaching a maximal difference between upper and lower sides after 24 h (Fig. 2). A four-fold transcript increase was detected on the lower side of inclined seedling stems 24 h after the onset of the inclination response in comparison to non-inclined control. In addition, transcript levels were strongly downregulated in needles and roots after inclination.

**Figure 3:**
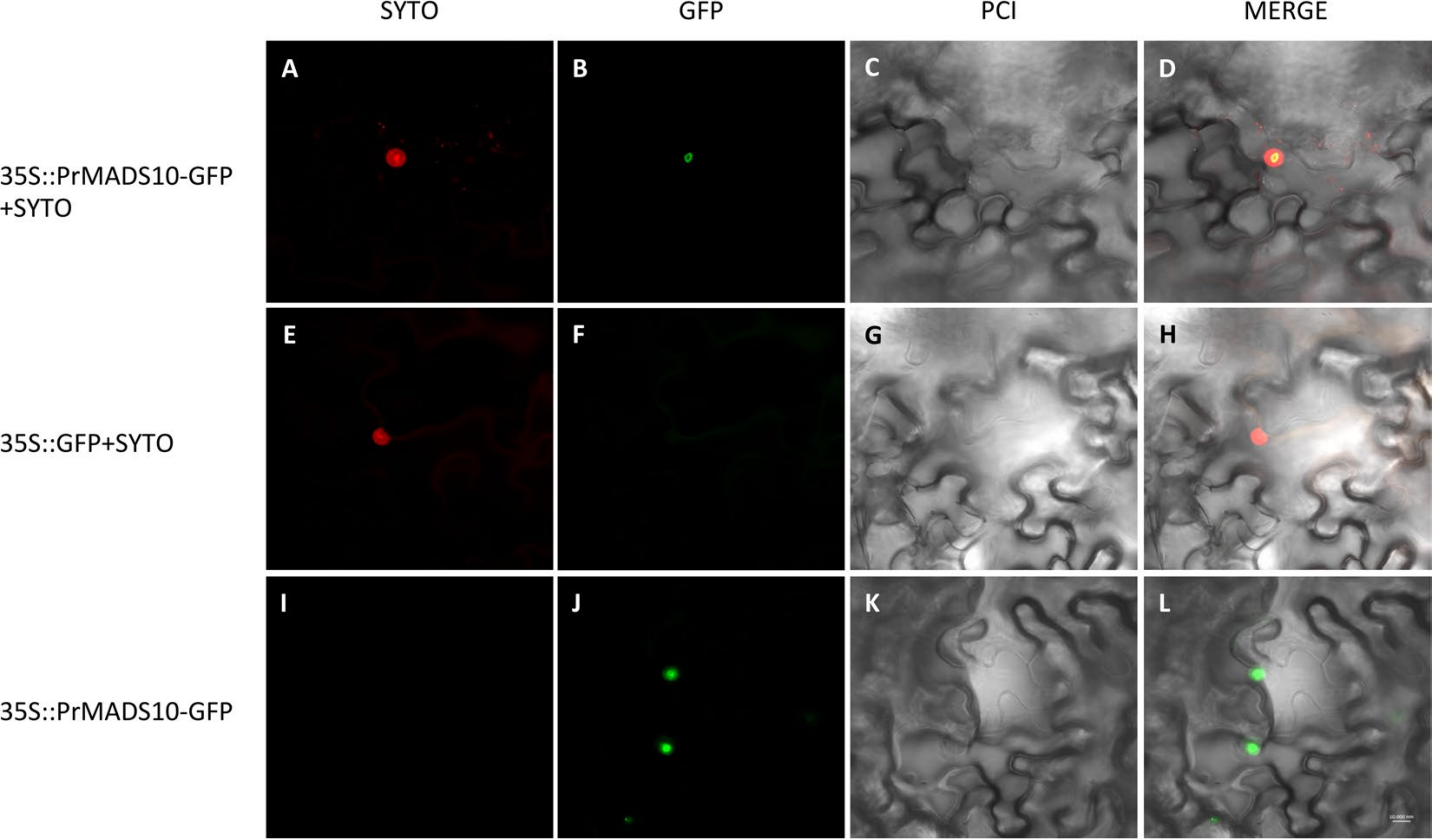
Subcellular localization of PrMADS10 fused to GFP (35 S::PrMADS10-GFP) in *Nicotiana benthamiana* leaves. SYTO83 was used to stain the nuclei. Merge: merging of GFP, SYTO83, and phase contrast image (PCI) images. Bar = 10 μm.

To determine the subcellular localization of *PrMADS10* tobacco leaves were agroinfiltrated^55^ (Fig. 3). *PrMADS10* fused to GFP co-localizes with SYTO83, a nuclear marker. This data reveals *PrMADS10* is a nuclear localized protein, consistent with its predicted function as a transcription factor based on protein sequence.

### Overexpression of *PrMADS10* in Arabidopsis alters lignin and flavonoids accumulation

**Figure 4:**
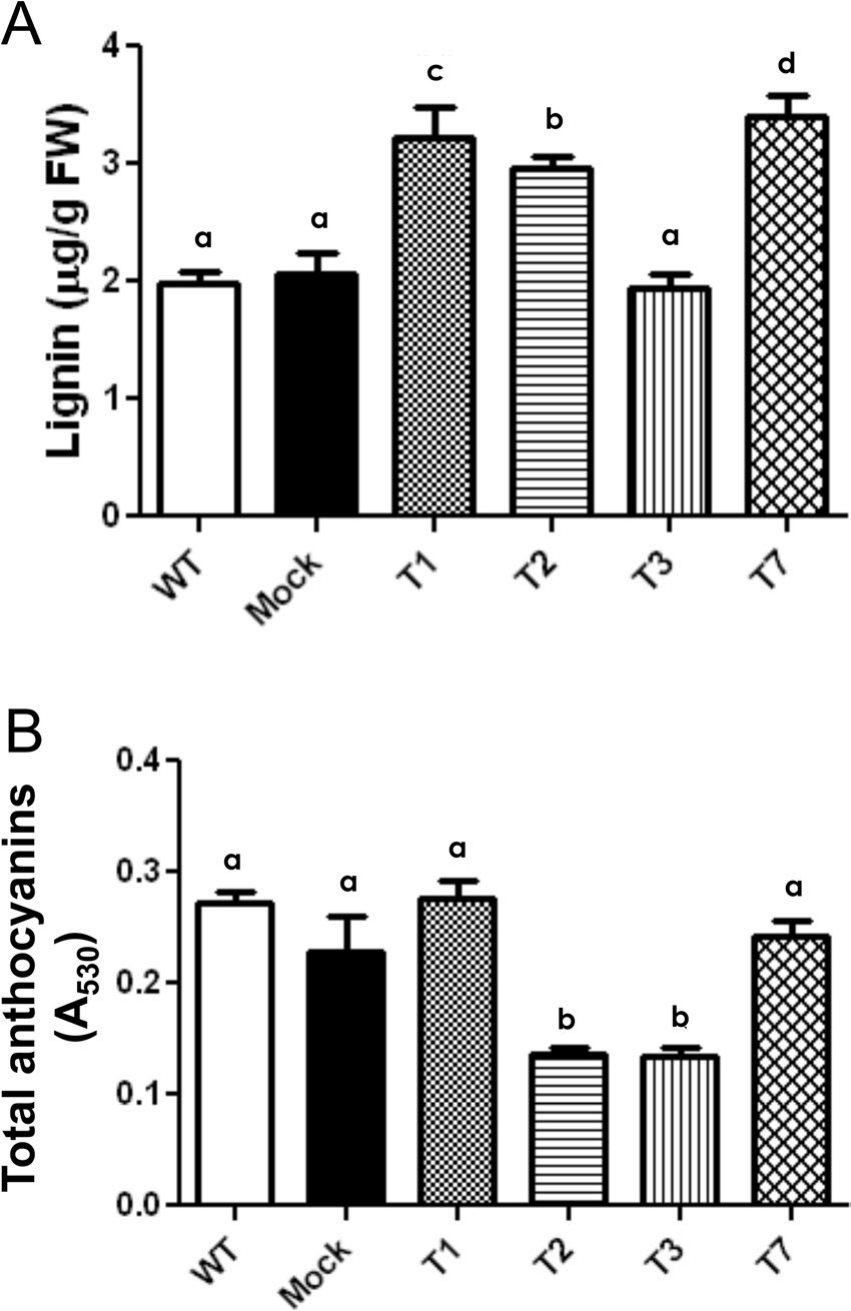
Quantification of lignin (**A**) and anthocyanins (**B**) in T3 Arabidopsis plants transformed with 35 S::PrMADS10. Data correspond to mean ± SE of three biological replicates per transgenic line of plants overexpressing *PrMADS10*, and different letters indicate statistical differences (*p* ≤ 0.05). Analysis of variance (ANOVA) and the t-student test was used.

The CDS of radiata pine *MADS10* gene was isolated and introduced into pBI121 binary vector under the control of CMV35S promoter. Homozygous Arabidopsis T3 transgenic lines were obtained and used for performing morphological and transcriptional analyses. Four different *PrMADS10* over-expressing lines were obtained (Suppl. Fig. 1). The evaluation of plant morphology and the weight of one hundred seeds indicated no changes between transgenic and control lines, however the length of rosette leaves were smaller in the transgenic lines (Suppl. Fig. 2). At the same time, lignin content in three of the transgenic lines was 30% higher than wild-type plants and Arabidopsis plants transformed with empty-vector (control), while one line showed no difference compared to controls (Fig. 4A). In addition, similar total area was observed when stem cross-sections from transgenic Arabidopsis and WT plants were stained with phloroglucinol-HCl. But, in the case of hypocotyl, two of three transgenic lines showed smaller total area compared to wild-type. On the other hand, qualitative difference was found in stem and hypocotyl when lignin was detected by using Phloroglucinol-HCl (Suppl. Fig. 3A,B). Besides, anthocyanin content was significantly-reduced in two transgenic lines (Fig. 4B).

### Gene modulation in Arabidopsis overexpressing *PrMADS10*

**Figure 5:**
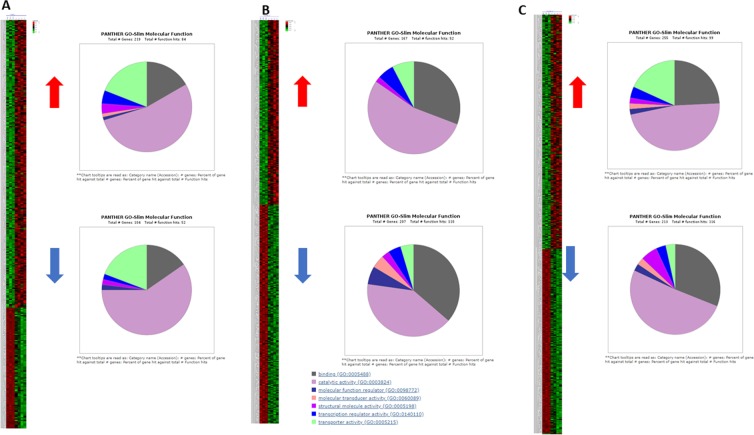
Unsupervised clustering with k-means and GO for molecular function of differentially expressed genes. (**A**) Cluster one has 339 genes in total with an homogeneity of 0.313, with 235 genes up and 104 down-regulated compared to control, and gene ontology classification for molecular function. (**B**) Cluster two has 383 genes in total with an homogeneity of 0.122, with 167 genes up and 207 down-regulated compared to control, and gene ontology classification for molecular function. **C**. Cluster three has 498 genes in total with an homogeneity of 0.040, with 280 genes up and 218 down-regulated compared to control, and gene ontology classification for molecular function. Sub-clustering considering the analysis of the 1219 differentially expressed genes (absolute expression), where red is 0~2 and green 0~−2, K-Means from Expander was used.

*PrMADS10* is differentially-expressed in inclined pine stems^29^, and could modulate the expression of a series of genes. Microarray analysis was used to determine global changes in gene expression resulting from constitutive over-expression of *PrMADS10*. The AraGene-1_0-ST 90k chip, with 28,501 annotated genes was used for the analysis. The raw data was normalized and differential gene expression was determined using standard statistical procedures (see Materials and Methods). We identified 1,219 differentially expressed genes when comparing transgenic and wild-type plants (Suppl. dataset 1). *PrMADS10* over-expression induces 690 and represses 529 genes. All 1,219 differentially expressed genes were analyzed using hieratical clustering (Suppl. Fig. 4). Three sub-clusters are shown in Suppl. Fig. 4. A high consistency is observed both within each group of plants (either control or transgenic), and within the branches generated. Unsupervised grouping chooses clustering by k-means, and three clusters were resolved having the first 339 genes, the second 383, and the third 498 genes. The first cluster has 235 up-regulated genes as compared to wild-type plants, 219 of which could be classified using the Panther database. 84 genes have an annotation in the molecular function gene ontology, with the following categories found: catalytic activity (53% of the genes), transporter activity (23%), binding (14%), transcription regulator activity (9%), structural molecule activity (4%), and both molecular transducer activity and molecular function as regulator (1%). A total of 104 genes were down-regulated, 52 of which had gene ontology annotations in similar categories for the up-regulated genes, except for the absence of molecular transducer activity category (Fig. 5A). Additionally, GOrilla^56^ was used with all the data from the microarray and it was determined that molecular function was the most significant category for this cluster (Suppl. Fig. 5A).

The second cluster contained 167 up-regulated genes, 52 of which were annotated as follows: 57% were classified to catalytic activity, 30% to binding, 9% to transporter activity, and 4% to structural molecule activity (Fig. 5B). 207 genes were down-regulated, with 110 annotated in different gene ontology categories: catalytic activity (43%), binding (32%), molecular function regulator (8%), transporter activity (5%), transcription regulator activity (5%), molecular transducer activity (5%), and structural molecule activity (2%) (Fig. 5B). When GOrilla was used in this cluster the best significant group was biological process category (Suppl. Fig. 5B).

The third cluster grouped 255 up-regulated genes, where 99 had gene ontology annotations: 47% classified to catalytic activity, 25% to binding, 19% to transporter activity, 3% to transcription regulator activity, 2% to structural molecule activity, 2% to molecular transducer activity and 2% to molecular function regulator. Finally, this cluster contains 213 down-regulated genes and 116 showed annotations in the molecular function ontology: most of them were related to catalytic activity (50%) and binding (30%), followed by molecular transducer activity (9%), transporter activity (3%), transcription regulator activity (3%), molecular function regulator (3%), and structural molecule activity (2%) (Fig. 5C). When GOrilla was used in this cluster the best significant group was biological process category (Suppl. Fig. 5C). Interestingly, in this cluster several sub-categories associated to stress and secondary metabolite are the most representatives.

The fifty most-up regulated genes were listed in Table 1 (those with >2.6 fold change). *Phosphate starvation1* showed the highest differential expression (over 5-fold change), and other genes related to phosphate metabolism or transport are higher and within the most-up regulated genes. Interestingly, other genes and TFs are also up regulated by the over-expression of *PrMADS10* in Arabidopsis. TFs like basic leucine-zipper 48, homeobox protein 16 are predominant, as well as, MIR399C, MIR399D and MIR827a. Other proteins like Ring U/Box superfamily, glutathione-S-transferase, alpha/beta hydrolases, rhamnose biosynthesis, phospholipase D, and phosphoenolpyruvate carboxylase kinase are all more abundant when *PrMADS10* is over-expressed.

**Table 1:** List of 50 most up-regulated genes in *35S::PrMADS10* transgenic Arabidopsis plants using microarray analysis.

AGI number	Exp log2	Gene description
AT3G09922	5.237	induced by phosphate starvation1
AT4G24890	5.107	purple acid phosphatase 24
AT1G17710	4.609	Pyridoxal phosphate phosphatase-related protein
AT5G20790	4.160	Unknown
AT3G61410	4.097	Unknown
AT1G23110	3.997	Unknown
AT5G17220	3.879	glutathione S-transferase phi 12
AT2G11810	3.879	monogalactosyldiacylglycerol synthase type C
AT3G44510	3.823	alpha/beta-Hydrolases superfamily protein
AT5G62162	3.822	MIR399C; miRNA
AT1G08310	3.748	alpha/beta-Hydrolases superfamily protein
AT3G14790	3.628	rhamnose biosynthesis 3
AT2G34202	3.591	MIR399D; miRNA
AT5G08030	3.577	PLC-like phosphodiesterases superfamily protein
AT1G73220	3.515	organic cation/carnitine transporter1
AT2G04038	3.475	basic leucine-zipper 48
AT2G45135	3.473	RING/U-box superfamily protein
AT2G45130	3.428	SPX domain gene 3
AT3G25240	3.409	Protein of unknown function (DUF506)
AT4G40060	3.372	homeobox protein 16
AT3G05630	3.344	phospholipase D P2
AT3G04530	3.306	phosphoenolpyruvate carboxylase kinase 2
AT2G34210	3.276	Transcription elongation factor Spt5
AT1G67600	3.263	Acid phosphatase/vanadium-dependent haloperoxidase-related protein
AT3G59884	3.258	MIR827a; miRNA
AT4G36350	3.246	purple acid phosphatase 25
AT3G02040	3.226	senescence-related gene 3
AT5G03545	3.134	Unknown
AT4G12090	3.096	Cornichon family protein
AT1G19200	3.078	Protein of unknown function (DUF581)
AT3G03530	3.052	non-specific phospholipase C4
AT4G01380	3.036	plastocyanin-like domain-containing protein
AT5G44562	3.019	other RNA
AT3G25233	3.014	Unknown
AT4G36850	3.008	PQ-loop repeat family protein / transmembrane family protein
AT4G17220	2.982	microtubule-associated proteins 70-5
AT3G09285	2.939	Unknown
AT3G02550	2.928	LOB domain-containing protein 41
AT5G53048	2.913	other RNA

On the other hand, terpenoid cyclase, O-acyltransferase, and fatty acid reductase are the most down-regulated genes. Interestingly, several TFs like WRKY, MYB, and B-box type zinc finger proteins are also down-regulated, as are other genes like expansins, pectin lyase, small auxin up RNA (SAUR), S-adenosyl-L-methionine (SAM) dependent methyltransferase and 2-oxoglutarate dependent oxygenase (Table 2).

**Table 2:** List of 50 most down-regulated genes in *35 S::PrMADS10* transgenic Arabidopsis using microarray analysis.

AGI number	Exp log 2	Gene description
AT1G78950	−3.176	Terpenoid cyclases family protein
AT5G22490	−3.128	O-acyltransferase (WSD1-like) family protein
AT3G57460	−3.068	catalytics;metal ion binding
AT3G56700	−3.022	fatty acid reductase 6
AT2G40080	−2.912	Protein of unknown function (DUF1313)
AT5G22570	−2.837	WRKY DNA-binding protein 38
AT2G24850	−2.734	tyrosine aminotransferase 3
AT5G42900	−2.690	cold regulated gene 27
AT1G57750	−2.643	cytochrome P450, family 96, subfamily A, polypeptide 15
AT5G13330	−2.555	related to AP2 6 l
AT4G33790	−2.551	Jojoba acyl CoA reductase-related male sterility protein
AT1G57560	−2.547	myb domain protein 50
AT1G02450	−2.530	NIM1-interacting 1
AT5G28080	−2.521	Protein kinase superfamily protein
AT2G21140	−2.516	proline-rich protein 2
AT1G07050	−2.501	CCT motif family protein
AT1G17665	−2.482	Unknown
AT5G38000	−2.481	Zinc-binding dehydrogenase family protein
AT1G30040	−2.469	gibberellin 2-oxidase
AT5G26220	−2.457	ChaC-like family protein
AT2G23910	−2.353	NAD(P)-binding Rossmann-fold superfamily protein
AT3G44860	−2.328	farnesoic acid carboxyl-O-methyltransferase
AT3G28220	−2.299	TRAF-like family protein
AT4G33980	−2.276	Unknown
AT2G21660	−2.271	cold, circadian rhythm, and rna binding 2
AT3G07650	−2.258	CONSTANS-like 9
AT5G44568	−2.254	Unknown
AT5G20630	−2.206	germin 3
AT1G66380	−2.183	myb domain protein 114
AT4G38825	−2.174	SAUR-like auxin-responsive protein family
AT1G68050	−2.169	flavin-binding, kelch repeat, f box 1
AT5G45960	−2.143	GDSL-like Lipase/Acylhydrolase superfamily protein
AT3G46490	−2.137	2-oxoglutarate (2OG) and Fe(II)-dependent oxygenase superfamily protein
AT3G05770	−2.108	Unknown
AT4G25860	−2.097	OSBP(oxysterol binding protein)-related protein 4 A
AT3G20810	−2.095	2-oxoglutarate (2OG) and Fe(II)-dependent oxygenase superfamily protein
AT5G37970	−2.073	S-adenosyl-L-methionine-dependent methyltransferases superfamily protein
AT5G37940	−2.070	Zinc-binding dehydrogenase family protein
AT3G60160	−2.068	multidrug resistance-associated protein 9
AT2G40610	−2.064	expansin A8
AT1G55525	−2.063	other RNA
AT5G48250	−2.051	B-box type zinc finger protein with CCT domain
AT4G17470	−2.032	alpha/beta-Hydrolases superfamily protein
AT1G28050	−2.016	B-box type zinc finger protein with CCT domain
AT1G14250	−1.992	GDA1/CD39 nucleoside phosphatase family protein
AT5G01900	−1.965	WRKY DNA-binding protein 62
AT3G59270	−1.964	FBD-like domain family protein
AT5G25460	−1.949	Protein of unknown function, DUF642
AT5G63180	−1.902	Pectin lyase-like superfamily protein
AT1G75780	−1.882	tubulin beta-1 chain

### Networking interaction analysis in Arabidopsis overexpressing *PrMADS10*

**Figure 6:**
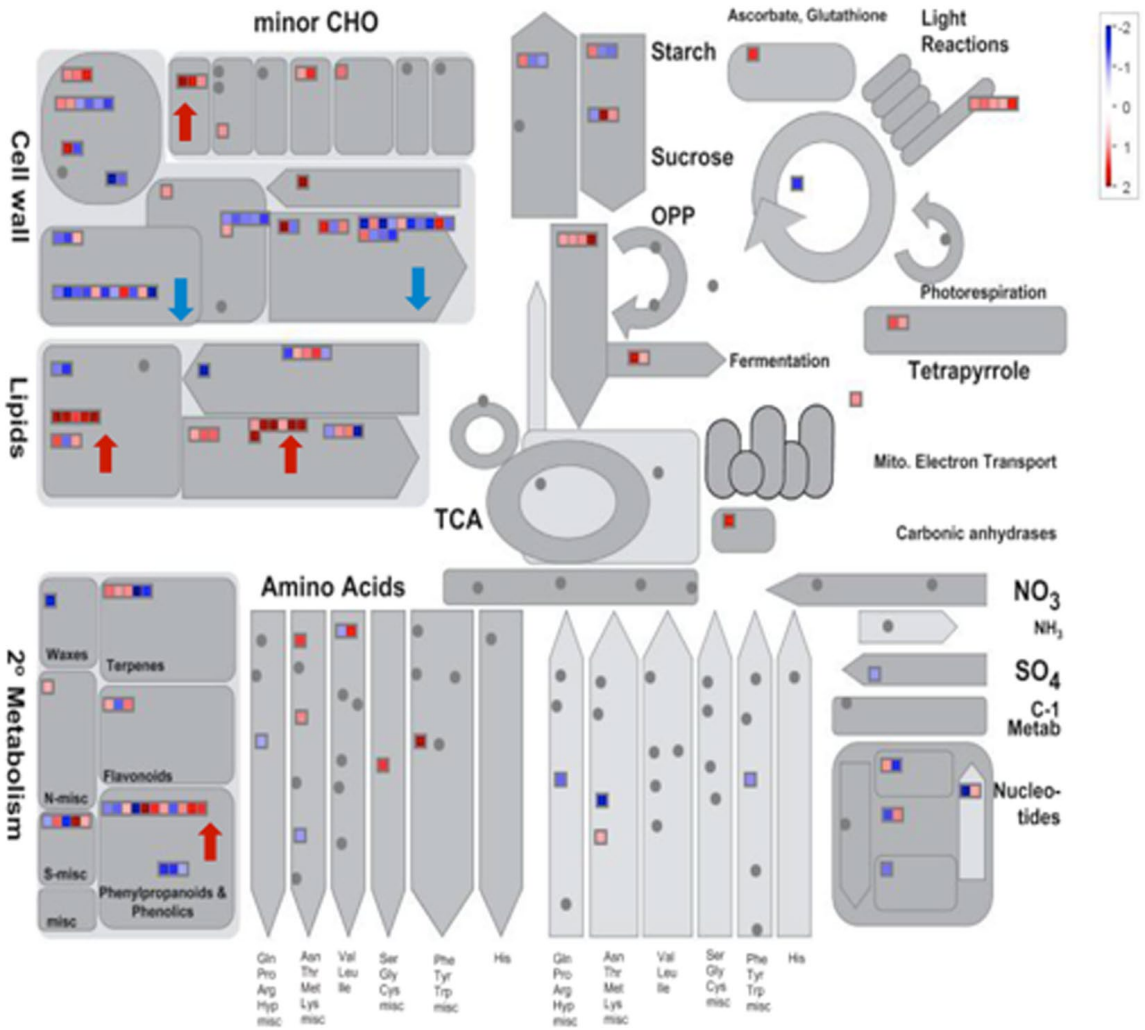
Metabolic overview using MapMan. The 1219 differentially-expressed genes were used to visualize the changes in metabolic pathways. One gene can have more than one classification. Up-regulated steps are represented in red and down-regulated steps in blue.

MapMan^53,54^ was used to visualize genes modulated by *35S::PrMADS10* in Arabidopsis. The terms for MapMan were assigned for 166 differentially-expressed genes; but due to a lack of matching MapMan terms, no functional assignment was done for the other 1,055 genes. The results obtained show a general view of different functional categories, which are being affected by *35S::PrMADS10*, such as major and minor carbohydrate, amino acid, nucleotide, fermentation, lipids, secondary metabolisms and cell wall (Fig. 6). Metabolism-related genes are further divided as 56 genes for cell wall, 30 for lipid, 29 for secondary, 12 for amino acid, 5 for light, 9 for major carbohydrate, 7 for minor carbohydrate, and a few for glycolysis, fermentation, tricarboxylic acid (TCA) cycle, S-assimilation, nucleotide metabolism, tetrapyrrole synthesis, or mitochondrial electron transport. When the phenylpropanoid pathway was analyzed, several genes were positively- or negatively-regulated (Fig. 6).

**Figure 7:**
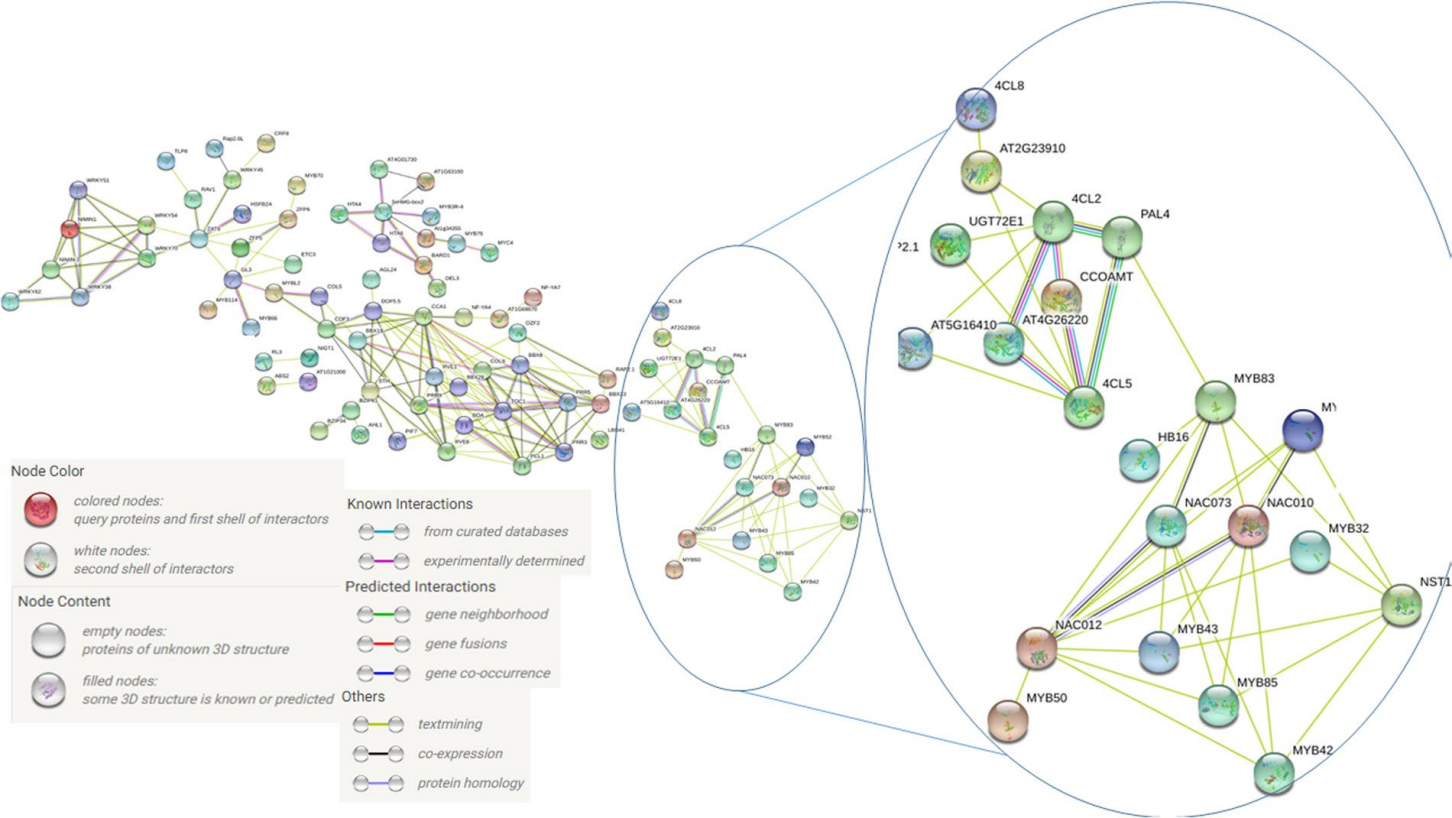
Network of gene interactions among genes differentially expressed using STRING. Genes used in the analysis were previously classified within the phenylpropanoid metabolic pathway and a series of TFs differentially expressed. Each node represents the interaction, described in the literature, between the proteins differentially-expressed in Arabidopsis over-expressing PrMADS10.

Differentially expressed TFs and phenylpropanoid pathway genes were selected from the microarray assay to perform a MapMan classification. The list of TFs and phenylpropanoid pathway´s genes were introduced in STRING in order to determine network interactions (Fig. 7). Genes without interactions were erased from the image. The interactions observed can be of different types, depending on the color of the line joining the spheres, and recognized as co-expression, co-localization and text-mining, and therefore it cannot be taken strictly as a direct protein-protein interaction. It can be observed that genes were clustered into different groups. TFs like MYB and NAC are included in one group (*MYB50*, *MYB52*, *MYB42*, *NAC010*, *NAC073*, *MYB43*, *MYB83*, *HB16*, *MYB85*, *MYB32*, *NAC012* and *NST2*), and *PAL*, 4-coumarate-CoA ligase (*4CL*), and caffeoyl-CoA O-methyltransferase (*CCoAOMT*) are in a different one. Interestingly, seven genes from the phenylpropanoid pathway are grouped and interconnected. MapMan showed that these genes (*PAL4*, *4CL8*, *4CL2*, *CCOAMT*, *4CL5*, CCR2; AT4G26220; UDP glucosyl transferase 72E1) are differentially expressed. The gene AT4G26220 has been reported as a probable *CCoAOMT7* (amplified view of Fig. 7).

In a small network interaction, MYC4 and MYB76 are grouped to other TFs. At the same time, LHY is related to AGL24, TOC1 and CCA, and indirectly to DOF, PRR5 or PRR9. Finally, a zinc transporter protein correlates with WRKY70, which has direct interactions with WRKY38, WRKY51 and WRKY54.

## Discussion

The importance in flower development was the initial interest to study MADS-box genes in plants^33^. However, a MADS-box gene (PdPI) was isolated from *Populus deltoides*, and it is expressed during flower development and in different vegetative organs. The evidence suggests that PdPI plays multiple functions in the development of this species^57^. In radiata pine, these TFs are expressed in male and female strobiles, for example *PrMADS3* (AGL6-like) is transcribed in the primordium of the acule^47^. Even though evidence defines roles for MADS-box TFs in flowering and other reproductive processes, their expression in roots, leaves and stems^33^, like in the case of *PrMADS10*, suggest another type of function in these other organs, perhaps expressed in response to stress.

In a first effort to understand the role that this MADS-box gene plays in radiata pine, the full length of *PrMADS10* was obtained by RACE. Deduced amino acid sequence was aligned with MADS-box proteins from different species available in Genbank database. PrMADS10 contains the typical MADS-box conserved domains: MADS-box, Intermittent, K-box and C-terminal^58^. According to Shore and Sharrocks^43^, a conserved sequence of 56 amino acids is characteristic in this domain, where 16 of which are identical in all family members. Structural features can also be observed in the MADS-box domain^44^, and the region of DNA responsible for protein interactions and dimerization^43^.

Phylogenetic analysis of PrMADS10 showed a distribution of sequences grouped into two main clades, with the sequences of interest being clustered in clade II. In this clade, all proteins have a MIKC-like structure^59,60^. In the phylogenetic tree, PrMADS10 is closely related to PaMADS1 from *P. abies* (Fig. 1B). PaMADS1 is expressed mainly in female and male pine cones, controlling the development of these tissues^61^. In addition, protein sequences of interest are also associated with a sub-clade classified as similar to SVP, AGL and MADS, such as PtSVP (*P. trichocarpa*) and AtSVP (*A. thaliana*), AtAGL24 (*A. thaliana*), StMADS16 (*S. tuberosum*) and PtMADS1 (*P. tomentosa*). PtSVP is a protein from the SVP (Short Vegetative Phase) family, and is very closely-related to AtSVP, which is a negative floral regulator^62^. This sequence is conserved in both angiosperms and gymnosperms. Similarly, AtAGL24 promotes the identity of the inflorescence in Arabidopsis whose expression occurs at the apex of the buds at the time of floral transition^63^. Most of the genes near to PrMADS10 were named orphan genes, because StMADS11, StMADS16, AtAGL24, and AtSVP are expressed in vegetative tissues, like vascular cambium region^64^, and having the same expression pattern than PrMADS10. Thus, the phylogenetic evidence suggests that PrMADS10 could be involved in floral development, but not observable in one-year-old seedlings. Nevertheless, this sequence was isolated from the lower side of pine stems, shortly after tilting^29^. This implies that its role in pine is related to the loss of verticality, modulating gene expression in vegetative tissues rather than in floral development.

*PrMADS10* was differentially expressed 2.5 hours after tilting and accumulation of transcripts was preferentially observed on the lower side of bent young pine stems^29^. The result suggests that expression of *PrMADS10* is temporally and spatially regulated in pine stem sections. The change in expression levels for this transcription factor in pine stems may be important for control of metabolic pathways that modify cell wall structure and properties which impacts on wood anatomy. Different molecular events related to cell wall modifications in response to verticality loss, including changes in a large number of TFs have been reported^1,29^. The work of Allona^1^ and Ramos^29^ were complementary, as both report new genes that respond to tilt, albeit there is no mention concerning the differential expression of MADS-box TFs in the former study.

Several different MADS-boxes have been described in radiata pine; PrMADS4 to 9 were detected in vegetative shoots, floral organs and roots, with greater expression in young floral tissue compared to adult tissue^46^. PrMADS1 to 3 could be involved in regulating female reproductive structures^47,48^ and cone development^49^. The expression of MADS-box genes during flower development and vegetative organs have also been reported in *P. deltoides*^57^. In contrast, Cseke^44^ reported PTM5 which is specific to vascular tissue and expressed in differentiated primary-secondary xylem and phloem. Therefore, although MADS-box genes are associated with flowering, other tissues like stem can be the tissue where this transcription factor can be expressed, which seems to be the case of *PrMADS10*, and more likely involved in stems modulating the response to inclination.

The AraGene microarray shows that *35S::PrMADS10* differentially regulates the expression of 1,219 genes when heterologously-expressed in Arabidopsis. *PrMADS10* induces the expression of 690 different genes and down-regulates that of 529. Aswath and Kim^33^ state that the constitutive expression of MADS-box genes into tobacco or Arabidopsis plants has proved to be a useful tool to analyze gene functions, as shown by the characterization of SAG1 from the conifer black spruce (*Picea mariana*), an homologous to AGAMOUS^65^. The overexpression of SAG1 produces homeotic floral conversion in transgenic Arabidopsis (from sepal to carpel and petal to stamen). On the other hand, overexpression of a MADS-box from *P. tomentosa* (PtAP3) in tobacco plants causes a fast growth and early flowering phenotype^66^^.^

The *35S::PrMADS10* transgenic lines of Arabidopsis did not show any discernible phenotypic changes, including flowering time, even though the transgene was over-expressed by at least 20,000 fold in all 4 lines obtained. The first 50 most-overexpressed genes were those involved in phosphate starvation, as well as, TFs and sucrose synthase 4 (SUS4) (Table 1). Unexpectedly, genes for phosphorous starvation were the most differentially-expressed. Pi was not a limiting factor in our assays, yet some reports indicate that Pi starvation is under the control of TFs required to maintain phosphorus homeostasis, which in turn is affected by environmental stress^67–71^.

Two genes, a multigenic AtSUS isoforms (SUS1–6) that synthesize monosaccharides for cellulose and starch biosynthesis^71^, and rhamnose biosynthesis 3 (RHM3), providing rhamnose for the synthesis of the cell wall^72^, are also up-regulated by *PrMADS10*. Basic chitinase is also up-regulated, which is a protein leading to inhibition of seedling growth^73^. In addition, the gene microtubule-associated protein 70-5 is positively-regulated by *PrMADS10*. This gene is essential for defining SCW polymers and is expressed in the cellular cortex of wood-forming cells, influencing the pattern of SCW thickenings in tracheary elements^74^. These results suggest that *PrMADS10* positively regulates several genes involved in wood formation, such as secondary metabolism, sugar synthesis or remodeling of cell wall components, as shown in the MapMan analysis (Fig. 7).

The accumulation of lignin is increased by 30% in the T3 transgenic lines, concomitant with the differential expression of genes involved in phenylpropanoid pathway, where a few genes from the pathway are positively-regulated by *PrMADS10*. Interestingly, *Pinus taeda* MYB4 has been reported as a positive regulator in lignifying cells^75^. Moreover, MYBs have also been related to cell wall metabolism, and have been shown to be the main master switches of SCW biosynthesis in different species^13,76,77^. More recently, the biosynthesis of cellulose/xylan/lignin is triggered by MYB46/83 and MYB85^78^. Both MYBs (MYB83 and MYB85) genes are up-regulated in our experiment, which indicates that *PrMADS10* can induce the expression of both TFs, thus enhancing the accumulation of lignin. A full network of protein-protein relationships reported by STRING indicates that these TFs are also associated with NAC. Moreover, the network correlates the interaction between MYBs and NACs with phenylpropanoid genes, such as *CCoAOMT*, *4CL2*, *4CL5*, *PAL4* and *4CL8*. The co-expression of these groups of genes was reported when transcriptomic analysis was performed in Arabidopsis stem during development^79^. Additionally, in woody plants like eucalyptus and poplar, the over-expression of MYB216 increases the accumulation of transcripts of 4CL5 and PAL4^80,81^. The knock-out for *PtMYB156* in poplar showed an increment in the accumulation of *4CL5* transcripts indicating a selective regulation of phenylpropanoid genes^82^.

TFs like NAC and MYB are preferentially co-expressed in xylem in *Picea glauca*^83^ and *Pinus pinaster*^84^, suggesting that both integrate a network which takes part in the development of complex wood traits, and as regulator for the synthesis of phenylalanine. In our assay, *NAC10*, *NAC073* and *MYB52*, *MYB42*, *MYB85* TFs were differentially expressed, and also showed relationships with other NACs and MYBs in the STRING network. Coincidently, several MYB TFs were characterized from radiata pine seedlings and are differentially-expressed on both sides of an inclined stem, yet preferentially on the upper side^85^. Whether NAC is modulating the expression of MYB and NAC genes, or if PrMADS10 could play a central role in this interaction in response to bending in pine, is an open question that requires further analysis.

The most regulated genes in the compression side of adult radiata pine tree trunks are those like: cell division, cellulose biosynthesis, lignin deposition and microtubules^86^. Genes like tubulin beta, sucrose synthase, proline-rich protein, and pectin lyase-like were differentially-expressed in *35S::PrMADS10* Arabidopsis, as well as, in adult pine trees^86^. Also, tubulin alpha chain, the cellulose synthase gene family, sucrose synthase and expansin were preferentially-expressed in *Pinus taeda* differentiated xylem^87^. These genes are active in some way during the process of cell wall remodeling, and are also differentially-expressed in *35S::PrMADS10* transgenic Arabidopsis.

Genes from the phenylpropanoid pathway are also differentially-regulated in *35S::PrMADS10* plants. The increase in *COMT* transcript levels was similar to that observed for *PAL*. If *COMT* levels increase, the amounts of synapyl alcohol should be modified, which is the last step for lignin (syringyl) synthesis. Similarly, if *COMT* and *CAD* transcript levels increase, they could modify the amounts of synapyl and p-coumaryl alcohols, the final step for the synthesis of lignin (syringyl and p-hydroxyphenyl, respectively). This is corroborated by previous studies, which suggest that the level of *C3H* transcripts could be determinant in the flow of metabolites towards lignin production, and the transcripts of *CCoAOMT*, *CCR* and *CAD* are modulated according to the metabolic demand^88^. Similar findings were reported in inclined radiata pine stems^29^. The net result of increasing the level of transcripts of these genes is an increment in flux through the monolignol portion for the lignin biosynthesis pathway^75^. Patzlaff^75^ reported that *PtMYB4* recognizes AC elements of the *PAL* promoter and genes encoding lignin biosynthesis enzymes are altered after overexpressing the *PtMYB4* gene in transgenic tobacco plants. In addition, the deposition of lignin is increased, and spreads to other types of cells that normally do not lignify, showing that *PtMYB4* was sufficient to induce lignification in a heterologous system. In contrast, overexpressing *PtrMYB3* from populus in Arabidopsis led to an increase in the deposition of the three main polymers of cell wall^89^. A 30% increment in lignin was observed in *35S::PrMADS10* lines, suggesting stimulation towards the lignin biosynthesis pathway. Besides, when one-year old pine seedlings were tilted, lignin accumulation and wall thickening were observed after 15 days^90^. This suggests that remodeling of the cell wall could initially be regulated by *PrMADS10* after tilting.

## Methods

### Materials and Methods

One year-old half-sib *Pinus radiata* D. Don (radiata pine) seedlings were grown in a local nursery from seeds obtained from an open-pollinated population (half sibs). Seedlings of around 30 cm tall were maintained at 20 °C, and following the protocol established previously^29,91^. Nine inclined seedlings were collected at each sampling time; their stems were cut into different parts along the longitudinal axis into lower and upper sides, pooled, immediately frozen in liquid nitrogen and stored at −80 °C until RNA extraction. Additionally, ‘non-inclined’ seedlings were sampled as control. Previous inclination procedure was followed^29^.

### Gene cloning and vector construction

*Pinus radiata* MADS10 (*PrMADS10*) was cloned from a cDNA library constructed from the stems of young seedlings exposed to inclination at different times^29^. The *PrMADS10* ORF was amplified and cloned following the RACE strategy, and using RNA from inclined stems (BD Smart RACE cDNA Amplification Kit, Clontech, USA). The following primers: PrMADS10-F 5′-CAGATCTCCGTCGGCAGTTAAAGGAAC-3′ and PrMADS10-R 5′-GGCTGCGAAGATAACCCTAGATGCAAG-3′ were used to obtain 3′ and 5′ sequences, respectively. PCR conditions for amplification was: 30 cycles each of 1 min at 94 °C, 1 min at 66 °C, and 3 min at 72 °C. A final 20-extension step of min at 72 °C was performed.

*PrMADS10* was cloned into the pBI121 binary vector, using forward (5′-GGATCCATGGCCGGCGAGAAAAGAAAGAT-3′) and reverse (5′-GAGCTCAATCTTTGATTCGGACGACTGT-3′) primers, designed to include BamHI and SacI restriction sites, respectively. The reverse primer had the stop codon deleted. The final construction, denominated *35S::PrMADS10*, was confirmed by sequencing.

For the construction of the PrMADS10-GFP fusion, the *PrMADS10* CDS was amplified with PrMADS10FL-F 5′-CACCATGGCCGGCGAGAAAAGAAAGAT-3′ and PrMADS10FL-R 5′-AATCTTTGATTCGGACGACTGT-3′ primers, without the stop codon and inserted into pENTR/D-TOPO vector (Invitrogen). Subsequently, the insert was transferred to the plant binary vector pKF7WG2 by recombination using Gateway® LR Clonase^TM^ II Enzyme Mix kit (Invitrogen) following manufacturer’s instruction. The 35 S::PrMADS10-GFP construct was used for subcellular localization studies. All clones used were confirmed by sequencing.

### Subcellular localization in tobacco leaves

Gateway® LR Clonase^TM^ II Enzyme Mix kit (Invitrogen) was used to perform a 35S:*PrMADS10*-*GFP* construction. Manufacturer’s instruction was followed for recombination.

After two days of cultures *Agrobacterium* was collected and centrifuged at 6000 x g for 10 min at 4 °C. The LB medium was supplemented with three antibiotics: gentamycin (100 μg/ml), rifampicin (10 μg/ml) and spectinomycin (50 μg/ml). Pellet was re-suspended in distilled and sterile water. Young tobacco leaves were infiltrated on the abaxial side, and were analyzed after three days post-infiltration. Syto® 84 Orange Fluorescent Nucleic Acid stain (Thermo Scientific) was used to label the nucleus. Subcellular localization of PrMADS10 in transient transformed leaves were analyzed through tissue visualization under a confocal fluorescence microscope (Carl Zeiss Confocal microscopy LMS 700) employing phase contrast image. Bar represent 10 μm.

### Stable arabidopsis transformation

Columbia ecotype (Col-0) of *Arabidopsis thaliana* (L.) Heynh plants were transformed using floral dip method^92^. Germinated seeds were placed in vessels containing rock wool and embedded in a hydroponic medium. The plants were maintained at 25 °C in the growth chamber with a long day photoperiod regime (16 h light/8 h dark). In the T2 generation, lines with single T-DNA insertion site were selected based on the segregation of resistant and sensitive seedlings to glufosinate ammonium containing medium and verified by PCR with specific primers. Then, T3 homozygous lines for PrMADS10 construct were selected and used in the analyses.

### RNA extraction and quantitative RT-PCR (RT-qPCR)

Total RNA was extracted from radiata pine seedlings following the procedure described by Le Provost^93^. Integrity of RNAs was checked on agarose gels stained with GelRed (Biotium Inc.), and their concentration determined by a ND-1000 UV spectrophotometer (Nanodrop Technologies, Montchanin, DE, USA). cDNA synthesis was performed using First Strand cDNA Synthesis Kit (Fermentas Life Science, Glen Burnie, MD, USA).

Total RNA was isolated from *35S::PrMADS10* transgenic Arabidopsis, using the SV total RNA isolation system (Promega). Primers for quantitative real time-PCR (RT-qPCR) were designed using Beacon Designer v 2.0 software (Premier Biosoft, Palo Alto, CA, USA). All primers used in this work are listed in Table 1. YBR Green/ROX quantitative PCR (qPCR) Master Mix (2 × ; Fermentas Life Science) was used for all qPCR quantifications in a final volume of 20 μL following the manufacturer’s protocol. All experiments were run on a real-time Mx3000P PCR detection system (Stratagene, Cedar Creek, TX, USA). The instrument was set to measure SYBR green dye fluorescence at the end of each cycle. Initial primer concentrations were 250 nM for all reactions, and the cDNA template for each sample was synthesized using 1 μg of DNase-treated total RNA using a first-strand cDNA synthesis kit (Fermentas Life Science) according to the manufacturer’s instructions. The first-strand RT reaction product was diluted ten-fold, and 2 μL was used for each qPCR reaction. The cycle threshold (Ct) line was determined manually as the point where the R2 value for the standard curve reached its highest point^94^. Standard curves were determined in duplicate reactions from the dilution series of each amplicon. qPCR determinations were run in duplicate and values of each sample corresponded to a mean ± SE of three biological replicates. A melting curve analysis was performed for each set of primers in order to avoid non-specific amplification. The expression levels were normalized with the stable expression level of three housekeeping genes (Suppl. Table 1). The overexpression of the PrMADS10 gene was obtained from 3 biological replicates. Relative expression was calculated using primers for AtFbox, AtUbi10^95^ and AtPP2 as normalizing genes. Data were analyzed using the methods derived from the algorithm of Vandesompele^96^. Two-way ANOVA-LSD post hoc was used to determine the main effects of inclination and time of inclination exposure effect for each gene using Statistica for Windows (v. 7.0; StatSoft, Tulsa, OK, USA). Significant differences were inferred at P ≤ 0.05. Differences in PrMADS10 transgenic expression, lignin and anthocyanin quantification was analyzed by one-way ANOVA-LSD post hoc and the significant differences inferred at P ≤ 0.05.

### Gene expression using Affymetrix ATH1 microarrays

For microarray hybridizations, total RNA was processed using the GeneChip one-cycle target-labeling kit (Affymetrix). Biotinylated cRNA was synthesized from 5 μg of total RNA from Arabidopsis stems (three months old) using the Affymetrix IVT kit according to the manufacturer’s instructions. cRNA was used to hybridize ATH1 GeneChip expression microarrays. Three un-transformed plants were used as controls and four transgenic lines were considered as biological replicate. Affymetrix data were normalized in R (http://www.r-project. org/) using RMA^97^. For detecting differentially regulated genes, normalized log2-transformed data were analyzed using Rank product statistic, as described before^98–100^. The data was processed with bioinformatics tools available at the VirtualPlant web site (http://www.virtualplant.org).

For the heatmap, Expander 7.11 (http://acgt.cs.tau.ac.il/expander/) was used. K-means of 3 clustering were obtained from microarray data. The clusters were obtained with 50 max iteration used distance metric with Pearson correlation. The GO pie charts were obtained using PANTHER14.1 DB and graphic genic onthology was obtained using GOrilla^56^.

### Microarray data

Raw signal intensity values were first normalized with RMA method using affy package in R language^101,102^ and probes were mapped to Locus ids of Arabidopsis genome. Genes with at least 2-fold change and *p-value* < 0.05 in Rank product analysis were considered as differentially expressed. Microarray data was analyzed using K-means clustering from Expander 7.11 (http://acgt.cs.tau.ac.il/expander/).

### Functional classification based on MapMan

Gene expression data in a context of metabolic overview was visualized using MapMan (version 3.6.0RC) software^53,54^. MapMan uses a plant-specific ontology that classifies genes into well-defined hierarchical categories, denominated BINs and suBINS^103^. The diagram shows positively and negatively-regulated genes in red and blue, respectively. The data set obtained from microarray analysis (AraGene-1_0-ST) where compared to Ath_AGI_TAIR9_Jan2010 integrated in MapMan from TAIR.

### STRING interaction network

String is a database where known and predicted direct (physical) interactions, as well as, indirect (functional) interactions can be established based on co-expression, co-localization, or text-mining and others^104–106^. Differentially-expressed genes related to transcription factors and those genes involved in the synthesis of lignin were picked and the web server was interrogated in order to uncover potential protein-protein association networks. The database was interrogated for the last time on 28^th^ March (2019).

### Anthocyanin and lignin content

Leaf and stem samples from three months-old Arabidopsis were ground in liquid nitrogen and extracted overnight in 1.0 mL of 1% (v/v) HCl in methanol at 4 °C following a previous report^107^. Relative anthocyanin levels were determined by measuring the absorbance at 530 nm of the aqueous phase^108^. Lignin was extracted as described by Campbell and Ellis^109^. Samples from whole Arabidopsis stems were diluted in 1 M NaOH (1/3, v/v) and hydrolyzed. A colorimetric assay was performed using thioglycolic acid (Sigma-Aldrich), and the absorbance was measured at 280 nm. The results were expressed as μg lignin per gram of fresh weight (FW).

## Supplementary information

Supplementary dataset 1

Supplementary Figures and table
