# Supplementary material for: Induction of PrMADS10 on the lower side of bent pine tree stems: potential role in modifying plant cell wall properties and wood anatomy
Source: Sci Rep. 2019 Dec 12;9:18981. doi: 10.1038/s41598-019-55276-7 (PMC6908731; doi:10.1038/s41598-019-55276-7)
Supplement: Supplementary file 2 — Supplementary Figures and table [file 41598_2019_55276_MOESM2_ESM.docx]

**Induction of PrMADS10 on the lower side of bent pine tree stems: potential role in modifying plant cell wall properties and wood anatomy**

**Nicolás Cruz^1,3^**^♯^**, Tamara Méndez^1^**^♯^**, Patricio Ramos^1,4^, Daniela Urbina^1^, Andrea Vega^2^, Rodrigo A. Gutiérrez^2^, María A. Moya-León^1^, Raúl Herrera^1*^**

**
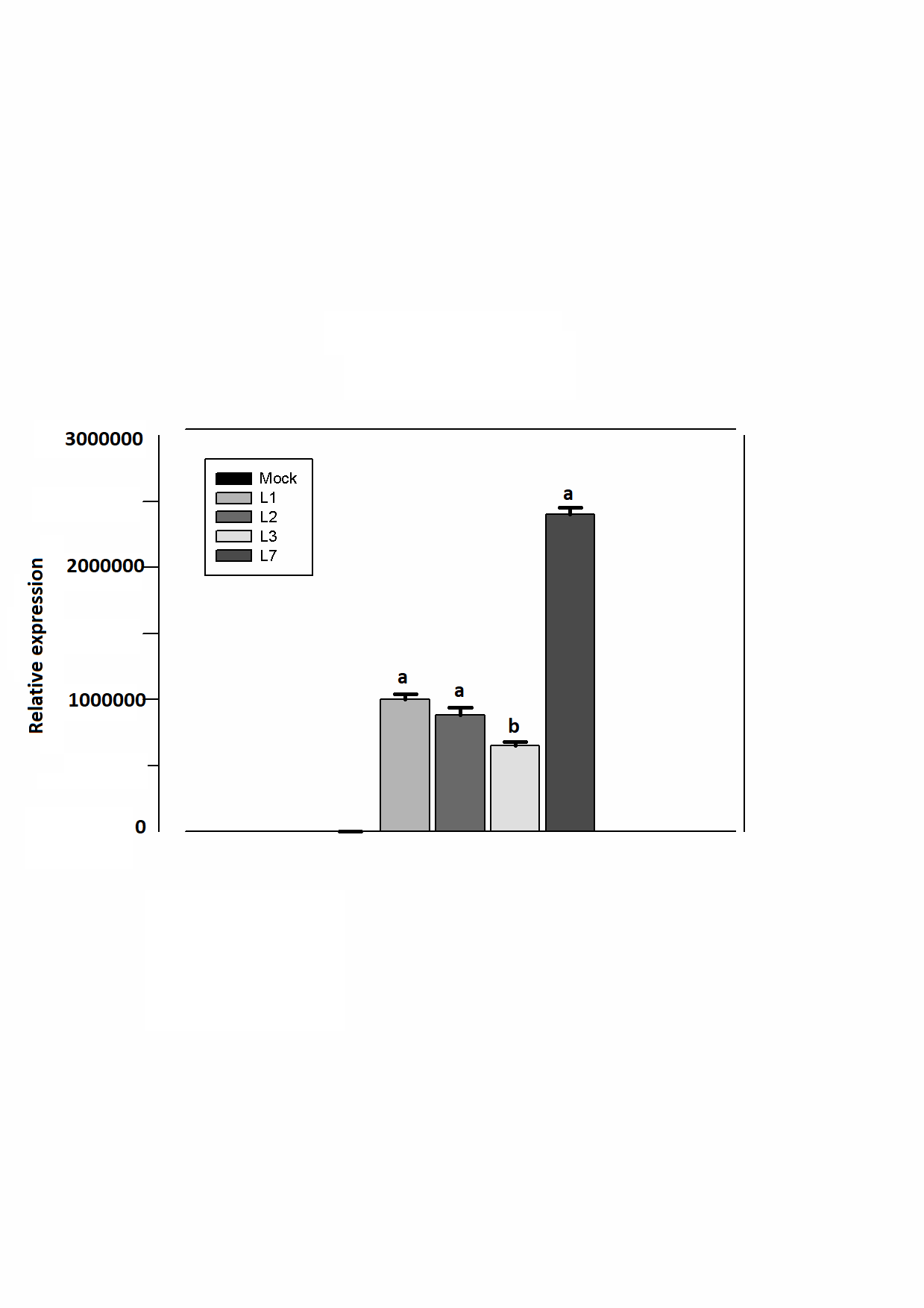
**

**Supplementary Figure 1. Transcripts levels of *PrMADS10* in Arabidopsis transgenic lines.** Four transgenic Arabidopsis lines were analyzed to determine the ***PrMADS10*** expression level. Data correspond to mean±SE of three biological replicates per transgenic line of plants overexpressing *PrMADS10.*

**
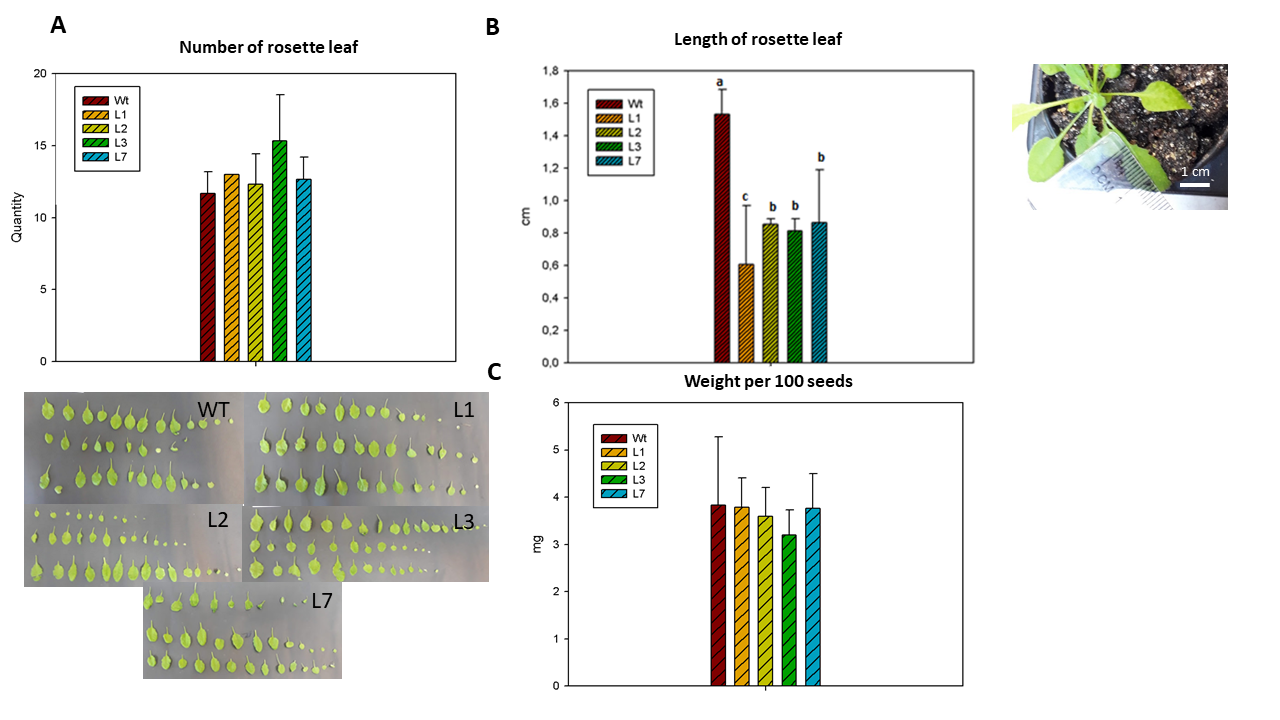
**

**Supplementary Figure 2. Transgenic plant morphology**. (A) Number of rosette leaf, (B) length of the rosette leaf and (C) weight of (100) seeds. Data correspond to mean±SE of three biological replicates per transgenic line of plants overexpressing *PrMADS10.*

**
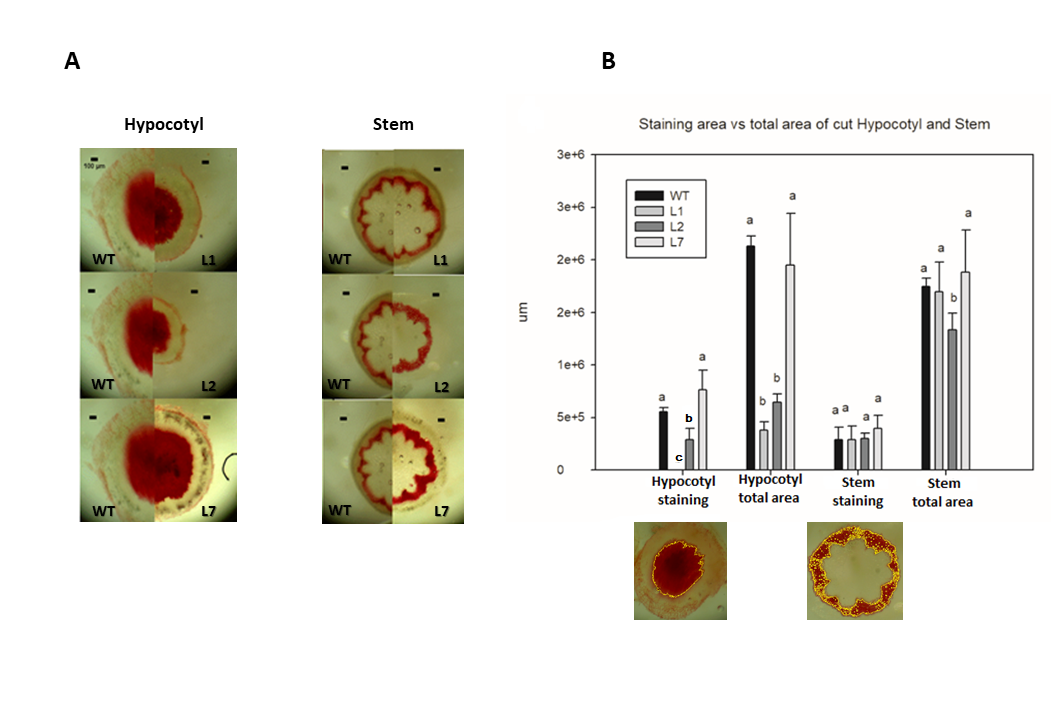
**

**Supplementary Figure 3. Wild type hypocotyl cut versus transgenic lines.** (A) Qualitative measure of wild type hypocotyl cuts and stem cuts versus transgenic lines, wildtype stem sections are show on the left side and transgenic stem sections are on the right. Basal stem (between rosette and first internode) and hypocotyl (from 5-6 weeks old plants after germination) were cut (scale in 100 μm).

(B) Area of staining and total area for hypocotyl and stem cross-sections in wildtype and transgenic lines.

**
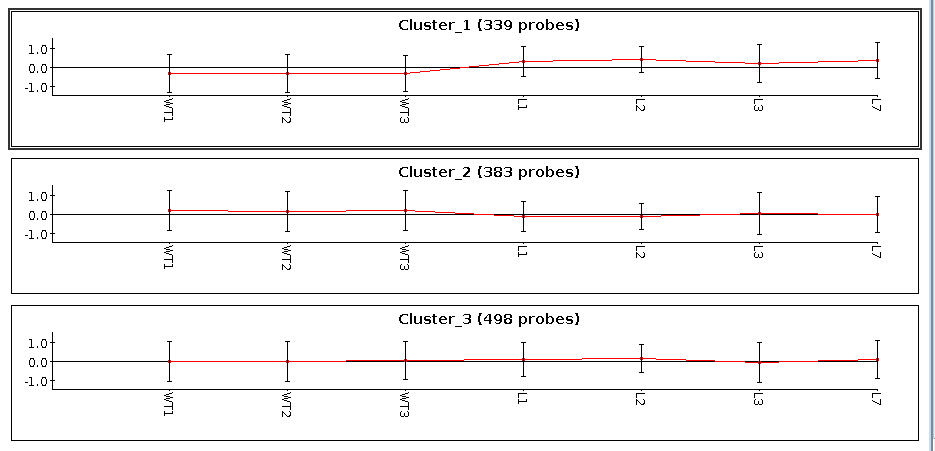
**

**Supplementary Figure 4. Clustering K-means.** With Expander generated 3 clusters, obtaining overall average homogeneity 0.143 an overall average separation -0.339. The separation of WT vs transgenic lines can be observed, considering all the up or down genes.

**
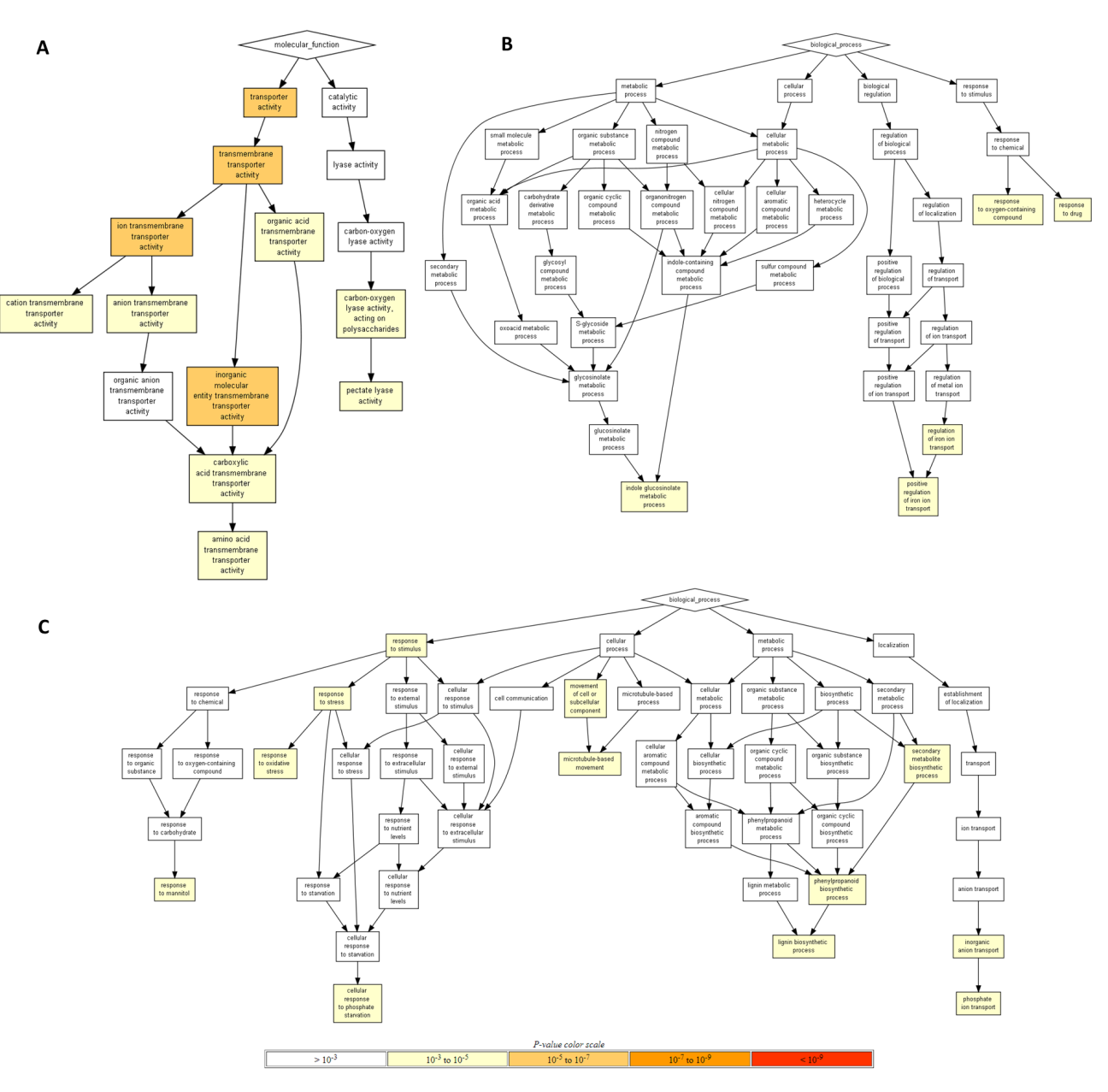
**

**Supplementary Figure 5. Gene Ontology (GO) according to GOrilla. A.** Three hundred and thirty nine genes corresponded to cluster one, having a major significance in molecular function category. **B.** Cluster two has 383 genes in total with a major category biological process. **C.** Cluster three has 498 genes in total and being biological process as the major category. The colour of the boxes corresponded to a p-value between >10^-3^ to 10^-9^.

**Suppl. Table 1**. List of primers used for RT-qPCR assays

| Target gene | Sequence Forward / Reverse (5'-3') | Expected fragment size | Specie |
| --- | --- | --- | --- |
| AtF-box-fw  AtF-box-rev | TTTCGGCTGAGAGGTTCGAGT  GAATTCCAAGACGTAAAGCAGATCAA | 61 | Arabidopsis |
| AtUbi10-fw  AtUbi10-rev | GGCCTTGTATAATCCCTGATGAATAAG  AAAGAGATAACAGGAACGGAAACATAGT | 60 | Arabidopsis |
| AtPP2a-fw  AtPP2a-rev | GGCAGAAGTTCGGATAGCAG  CAATGCAGATCTGACGTGCT | 59 |  |
| PrMADS10-fw  PrMADS10-rev | CATAAGTCGCCCATTGAAGG  GATCACGGAAGACGACGATT | 57 | Pine |
| Ge066D02-fw  Ge066D02-rev | TTTTAGGAAGAAGGGTGATTGACT  ATTAGAAACCAACGAGGCTGTC | 58 | Pine |
